# Effect of SGLT-2 Inhibitors on Prognosis in Diabetic Patients with Acute Myocardial Infarction: A Systematic Review and Meta-Analysis

**DOI:** 10.31083/j.rcm2505154

**Published:** 2024-05-06

**Authors:** Zhiwei Li, Anying Li, Dianhan Sun, Yusheng Shu

**Affiliations:** ^1^Graduate School of Dalian Medical University, Dalian Medical University, 116044 Dalian, Liaoning, China; ^2^Department of Thoracic Surgery, Subei People's Hospital of Jiangsu Province, 225001 Yangzhou, Jiangsu, China

**Keywords:** SGLT2, diabetes mellitus, AMI, prognosis, meta-analysis

## Abstract

**Background:**

The present meta-analysis aimed to examine the effects of sodium-glucose cotransporter 2 (SGLT2) inhibitors on the prognosis of diabetes patients who experienced acute myocardial infarction (AMI). This investigation encompassed an array of clinical endpoints, comprising cardiovascular death, myocardial reinfarction, all-cause mortality, major adverse cardiovascular events (MACEs), and rehospitalization.

**Methods:**

The study was conducted in accordance with the Preferred Reporting Items for Systematic Reviews and Meta-Analyses (PRISMA) guidelines. The PubMed, Cochrane Library, Embase, and Web of Science databases were searched up to October 2023. Studies reporting clinical outcomes in diabetic patients who experienced AMI and were treated with SGLT2 inhibitors (SGLT2-I) were included. Two researchers independently selected the studies and assessed the risk of bias in the included studies using the Cochrane risk of bias tool for Risk for Bias In Non-randomized Studies-of Interventions (ROBINS-I).

**Results:**

A total of 2450 publications were initially retrieved; ultimately, five studies involving 5398 patients were included in the meta-analysis. The analysis revealed that SGLT2-I were associated with significantly lower risks of cardiovascular death (odds ratio (OR), 0.34; 95% CI, 0.14–0.82) and all-cause mortality (OR, 0.54; 95% CI, 0.38–0.76). However, SGLT2-I did not lead to a significant decrease in the rate of myocardial reinfarction (OR, 0.91; 95% CI, 0.65–1.29). SGLT2-I did lead to a significant reduction in MACEs (OR, 0.59; 95% CI, 0.35–1.0), but there was significant heterogeneity among the included studies. SGLT2-I also led to a significant reduction in rehospitalizations (OR, 0.45; 95% CI, 0.26–0.76). There was significant heterogeneity in the analysis of rehospitalization, but the effect remained significant when we excluded the main sources of heterogeneity (OR, 0.35; 95% CI, 0.24–0.52).

**Conclusions:**

The pooled analyses revealed that SGLT2-I were associated with reductions in all-cause mortality, cardiovascular death, and rehospitalization. In the future, prospective studies with larger sample sizes are needed to confirm and refine these findings.

## 1. Introduction

Diabetes is a chronic metabolic disorder characterized by elevated blood sugar levels [1]. Acute myocardial infarction (AMI) is a sudden and life-threatening event resulting from the disruption of blood flow to the heart muscle [2]. Notably, in clinical practice, cardiovascular disease is most often the primary cause of mortality in patients with diabetes mellitus (DM) [3]. Patients with DM have worse prognoses after experiencing an AMI than patients without DM [4]. Therefore, proactive treatment should be given to diabetic patients after the occurrence of AMI.

Sodium-glucose cotransporter 2 (SGLT2) inhibitors represent a novel class of oral hypoglycemic agents that have demonstrated the ability to improve cardiovascular outcomes in patients with Type 2 diabetes mellitus (T2DM) and heart failure [5, 6, 7, 8]. Preclinical investigations have provided evidence that SGLT2 inhibitors (SGLT2-I) can mitigate acute myocardial I/R injury, reduce cardiac infarct size, improve left ventricular function, and decrease the risk of arrhythmias [9, 10]. In a clinical context, some studies have demonstrated that T2DM patients hospitalized for AMI who receive SGLT2-I treatment show significant reductions in inflammatory burden, arrhythmic burden and infarct size compared to patients not receiving SGLT2-I treatment, and this effect is unrelated to glycemic control [11, 12]. Thus, it is reasonable to examine the potential of SGLT2-I to improve outcomes in diabetic patients who experienced AMI.

Although previous studies have shown the impact of SGLT2-I on diabetic patients at high risk for cardiovascular disease, their effect on T2DM patients who experienced AMI remains unclear. Therefore, this meta-analysis aims to investigate the impact of SGLT2-I on the prognosis of T2DM patients who have experienced AMI.

## 2. Material and Methods

This study was conducted in accordance with the Preferred Reporting Items for Systematic Reviews and Meta-Analyses (PRISMA) guidelines [13]. Additionally, our study protocol was registered in PROSPERO (registration number: CRD42023458812).

### 2.1 Search Strategy

The PubMed, Cochrane Library, Embase, and Web of Science databases were comprehensively searched up to October 2023. The detailed search strategies for this meta-analysis are shown in **Supplementary Table 1**. Moreover, we searched the reference lists of the retrieved articles to identify any potentially eligible studies.

### 2.2 Inclusion and Exclusion Criteria

Studies were considered eligible for inclusion if they met the following criteria: (a) diabetic patients who experienced AMI; (b) patients treated with SGLT2-I after AMI; and (c) studies reported the primary or secondary outcomes.

The exclusion criteria were as follows: (a) duplicate articles; (b) abstracts, editorial comments, letters, case reports, reviews, or meta-analyses; (c) articles with titles and abstracts that were clearly unrelated to the topic of interest; (d) full-text articles not in English; and (e) articles with unavailable data.

Based on the inclusion and exclusion criteria, two researchers independently screened the titles and abstracts of the retrieved articles; then, they screened the full texts of the potentially eligible articles. Any disagreements or discrepancies between the researchers were resolved through consensus.

### 2.3 Risk of Bias Assessment

The two researchers independently assessed the risk of bias in the included studies using the Cochrane risk of bias tool for Risk for Bias In Non-randomized Studies-of Interventions (ROBINS-I). Any disagreements or discrepancies were resolved through discussion and consensus.

### 2.4 Data Extraction

The two researchers independently extracted the following data from the included studies: first author, publication year, study characteristics (country, study design, and study period), patient characteristics (including the number of patients, age, sex distribution, median follow-up time in years, left ventricular ejection fraction (LVEF), and the number of ST-elevation myocardial infarction (STEMI) occurrences), and outcomes (including cardiovascular mortality, rate of myocardial reinfarction, rate of rehospitalization, all-cause mortality, and incidence of major adverse cardiovascular events (MACEs)). MACEs were defined as a composite of all-cause mortality, non-fatal MI (NFMI), revascularization, cerebrovascular accident, and rehospitalization. When continuous variables were reported in the form of medians with ranges or interquartile ranges in the original studies, we converted them into means ± standard deviations through a previously validated mathematical method [14, 15]. Any discrepancies that arose during the data extraction process were resolved by consensus.

### 2.5 Statistical Analysis

Statistical analysis was performed in STATA 15.1 (StataCorp LLC, College Station, TX, USA). All outcomes were reported as odds ratios (OR) and 95% confidential intervals (CIs). To assess the level of heterogeneity among the studies, the chi-squared (χ^2^) test and inconsistency index (*$I^{2}$*) were utilized [16]. A *p* value < 0.05 for the χ^2^ test or an *$I^{2}$* value > 50% was considered to indicate significant heterogeneity. Notably, the presence of heterogeneity within specific findings prompted the utilization of a random effects model. To assess potential publication bias, a funnel plot was generated to facilitate an intuitive evaluation. In addition, we performed one-way sensitivity analyses to evaluate the influence of individual studies on the pooled results for outcomes with significant heterogeneity.

## 3. Results

### 3.1 Literature Search and Study Selection

**Fig. 1.**
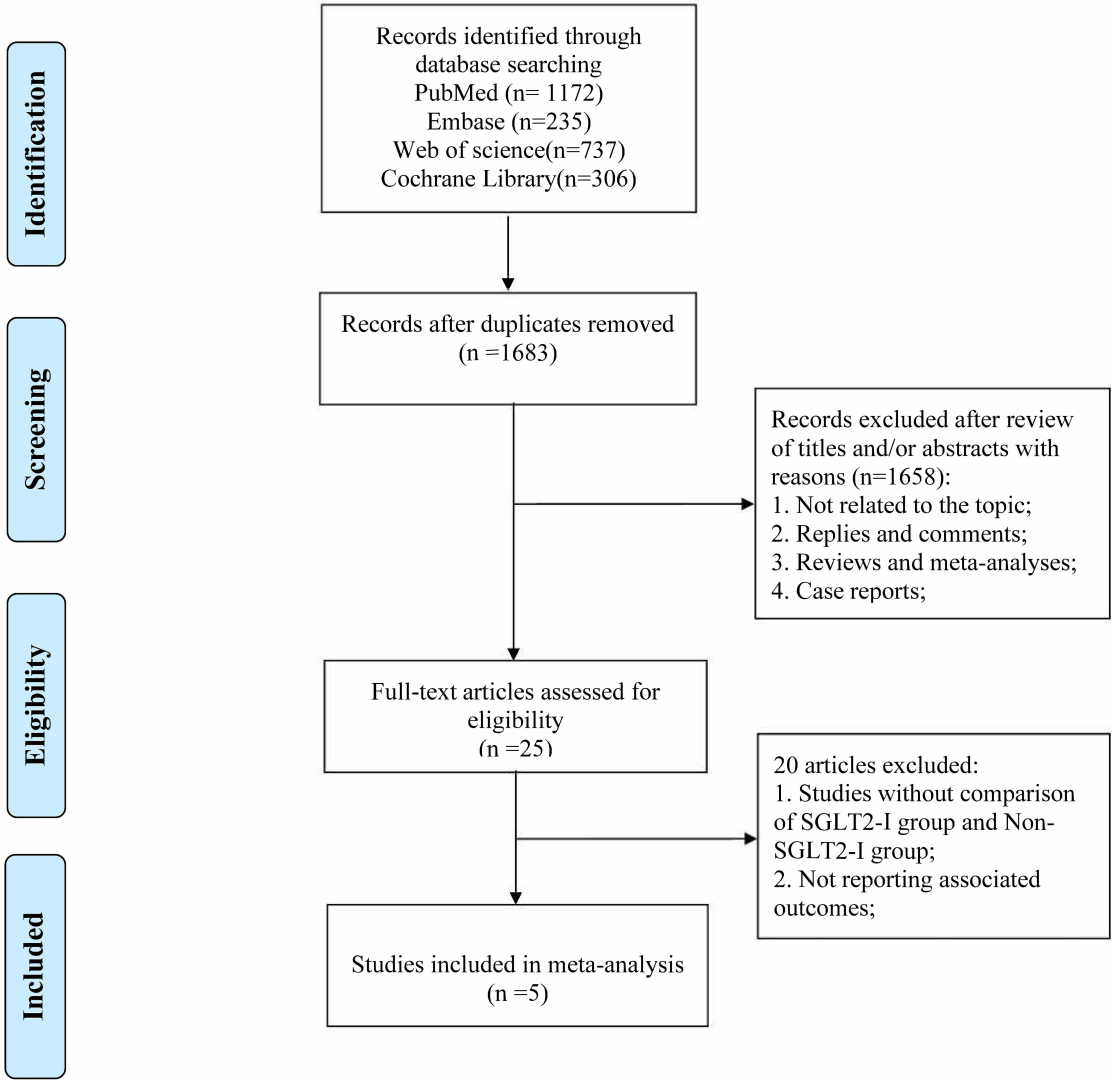
**The flow diagram of study selection**. SGLT2-I, sodium-glucose cotransporter 2 inhibitors.

The initial search yielded a total of 2450 publications; 1683 studies remained after excluding 767 duplicate studies. Following a review of the titles and abstracts, 1658 studies were excluded. The remaining 25 articles underwent a thorough evaluation of the full text, leading to the exclusion of an additional 20 articles for the following reasons: (1) studies lacked a comparison between the SGLT2-I group and non-SGLT2-I (non-SGLT2 inhibitors) group; and (2) did not report the outcomes of interest. Ultimately, 5 articles were eligible for this meta-analysis. A PRISMA flow diagram of the study selection process is shown in Fig. 1.

### 3.2 Study Description and Risk of Bias Assessment

A total of 5 eligible studies encompassing 5398 patients (1576 in the SGLT2-I group and 3822 in the non-SGLT2-I group) were included in the pooled analysis [17, 18, 19, 20, 21]. All included studies were retrospective in nature. Table 1 (Ref. [17, 18, 19, 20, 21]) presents a summary of the study and patient characteristics. The details of the risk of bias assessment of all eligible studies are provided in Table 2 (Ref. [17, 18, 19, 20, 21]).

**Table 1.** **Characteristics of patients included in the study**.

Author	Year	Origin	Study period	Design	Age (years, mean ± SD)		Male:Female		No. of patients		LVEF (%, mean ± SD)		STEMI		Median follow-up (years)
					SGLT2-I	non-SGLT2-I	SGLT2-I	non-SGLT2-I	SGLT2-I	non-SGLT2-I	SGLT2-I	non-SGLT2-I	SGLT2-I	non-SGLT2-I	
Young Sang Lyu [17]	2023	Korea	2016–2020	retrospective	59.11 ± 11.52	66.12 ± 10.86	150:36	422:171	186	593	51.07 ± 12.20	52.58 ± 11.40	100	227	0.99
Osung Kwon [18]	2023	Korea	2014–2018	retrospective	56.4 ± 11.3	57.6 ± 11.3	769:169	1482:394	938	1876	NA	NA	550	1137	2.1
Ting-Yung Chang [19]	2022	China	2016–2020	retrospective	66.1 ± 12.3	67.7 ± 11.9	50:16	95:37	66	132	52.0 ± 12.8	52.3 ± 10.6	NA	NA	1.96
Pasquale Paolisso [20]	2023	Italy	2018–2021	retrospective	66 ± 10.52	71.30 ± 13.38	90:21	405:130	111	535	48 ± 10	47 ± 11	52	257	2
Lipeng Mao [21]	2023	China	2017–2021	retrospective	61.97 ± 13.22	67.22 ± 12.15	209:66	451:235	275	686	49.67 ± 9.87	49.86 ± 9.10	167	398	1.48

STEMI, ST segment elevation myocardial infarction; LVEF, left ventricular ejection fraction; NA, not available; SGLT2-I, sodium-glucose cotransporter 2 inhibitors; non-SGLT2-I, non-sodium-glucose cotransporter 2 inhibitors; SD, standard deviation.

**Table 2.** **Risk of bias in included studies**.

Study	Bias due to confounding	Bias in selection of participants into the study	Bias in classification of interventions	Bias due to deviations from intended interventions	Bias due to missing data	Bias in measurement of outcomes	Bias in selection of the reported result	Overall assessment
Young Sang Lyu 2023 [17]	Moderate	Low	Low	Low	Moderate	Low	Low	Moderate
Osung Kwon 2023 [18]	Moderate	Low	Moderate	Low	Low	Low	Low	Moderate
Ting-Yung Chang 2022 [19]	Moderate	Low	Low	Low	Low	Low	Low	Moderate
Pasquale Paolisso 2023 [20]	Moderate	Low	Low	Low	Moderate	Low	Low	Moderate
Lipeng Mao 2023 [21]	Moderate	Low	Low	Low	Low	Low	Low	Moderate

### 3.3 Cardiovascular Death

**Fig. 2.**
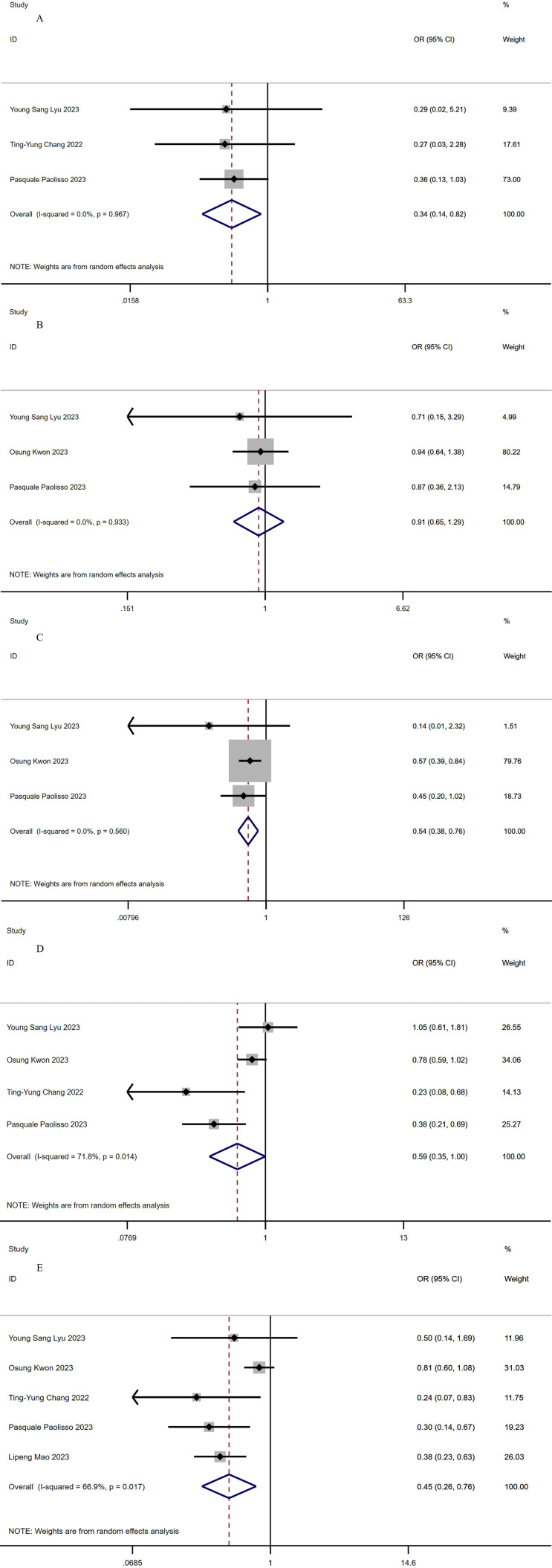
**Forest plots of outcomes**. (A) Cardiovascular death. (B) Myocardial reinfarction. (C) All-cause mortality. (D) MACEs. (E) Rehospitalization. MACEs, major adverse cardiovascular events.

**Fig. 3.**
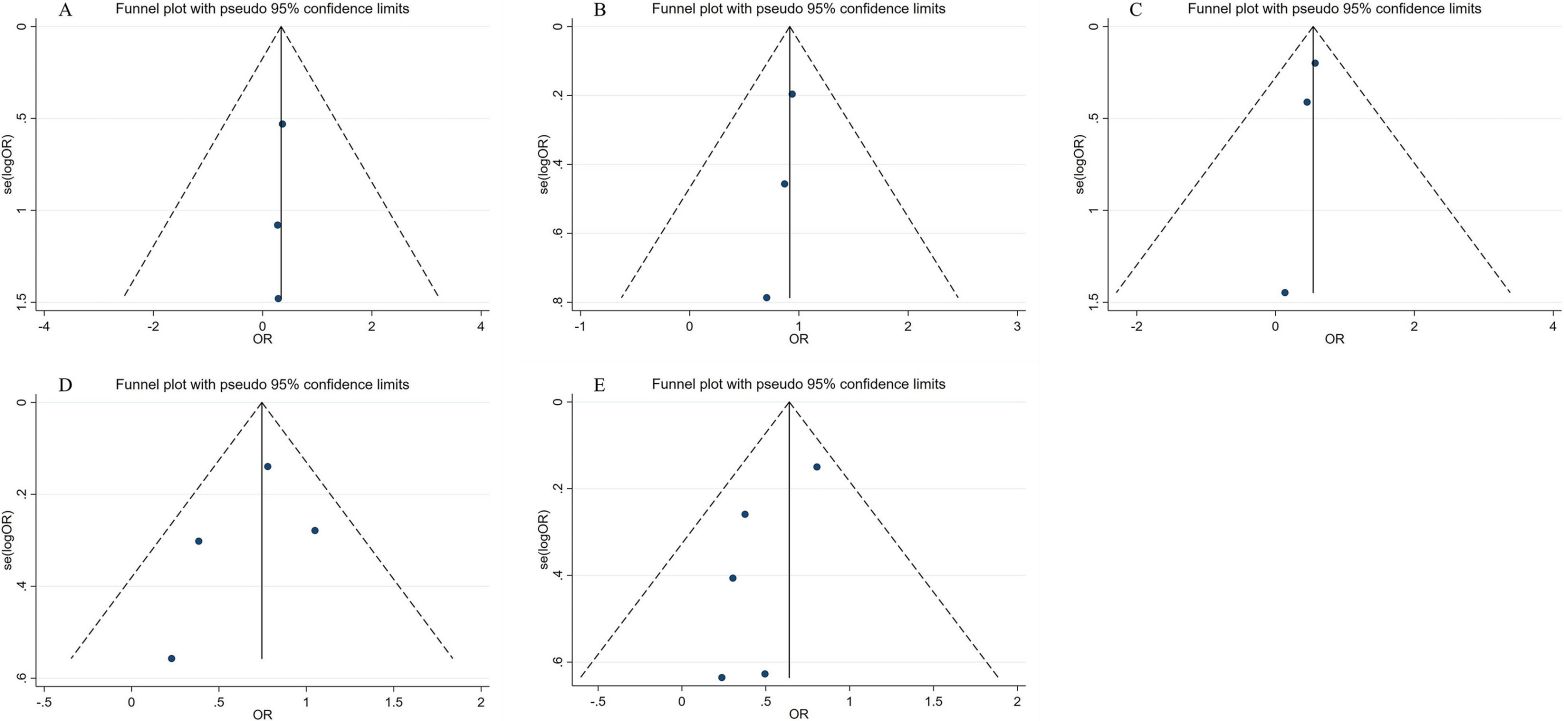
**Funnel plots of (A) Cardiovascular death, (B) Myocardial reinfarction, (C) All-cause mortality, (D) MACEs, and (E) Rehospitalization**. OR, odds ratio; MACEs, major adverse cardiovascular events

Three studies with a total of 1623 patients (363 in the SGLT2-I group versus 1260 in the non-SGLT2-I group) reported cardiovascular mortality [17, 19, 20]. No significant heterogeneity (*$I^{2}$* = 0%, *p* = 0.97) was observed. The utilization of SGLT2-I was associated with a lower risk of cardiovascular mortality compared to not using SGLT2-I (OR, 0.34 [95% CI, 0.14–0.82]; *p* = 0.017; Fig. 2A). Additionally, Fig. 3A illustrated that the funnel plot did not reveal any evidence of publication bias.

### 3.4 Myocardial Reinfarction

Three studies with a total of 4239 patients (1235 in the SGLT2-I group versus 3004 in the non-SGLT2-I group) reported myocardial reinfarction [17, 18, 20]. The pooled analysis revealed that the use of SGLT2-I did not yield a statistically significant reduction in the rate of myocardial reinfarction (OR, 0.91 [95% CI, 0.65–1.29]; *p* = 0.612; Fig. 2B). No significant heterogeneity was observed (*$I^{2}$* = 0%, *p* = 0.93). Furthermore, the assessment through the funnel plot did not indicate any presence of publication bias, as demonstrated in Fig. 3B.

### 3.5 All-Cause Mortality

Three studies with a total of 4239 patients (1235 in the SGLT2-I group versus 3004 in the non-SGLT2-I group) reported all-cause mortality [17, 18, 20]. The pooled results revealed a significant reduction in all-cause mortality in the SGLT2-I group compared with the non-SGLT2-I group (OR, 0.54 [95% CI, 0.38–0.76]; *p* = 0; Fig. 2C), and no significant heterogeneity was observed (*$I^{2}$* = 0%, *p* = 0.56). The funnel plot in Fig. 3C similarly demonstrates an absence of significant heterogeneity.

### 3.6 MACEs

Four studies with a total of 4437 patients (1301 in the SGLT2-I group versus 3136 in the non-SGLT2-I group) reported MACEs [17, 18, 19, 20]. The pooled analysis indicated that the group using SGLT2-I had a significantly lower rate of MACEs (OR, 0.59 [95% CI, 0.35–1.0]; *p* = 0.049; Fig. 2D), but there was significant heterogeneity (*$I^{2}$* = 71.8%,* p* = 0.01). Moreover, a visual assessment of the funnel plot indicated the presence of slight publication bias (Fig. 3D).

### 3.7 Rehospitalization

Five articles with a total of 5398 patients (1576 in the SGLT2-I group versus 3822 in the non-SGLT2-I group) reported data on rehospitalization [17, 18, 19, 20, 21]. The pooled results showed that the rate of rehospitalization was significantly lower in the SGLT2-I group than in the non-SGLT2-I group (OR, 0.45 [95% CI, 0.26–0.76]; *p* = 0.003; Fig. 2E). However, statistically significant heterogeneity was observed (*$I^{2}$* = 66.9%, *p* = 0.02). Furthermore, an analysis of the funnel plots indicated the presence of publication bias, as depicted in Fig. 3E.

### 3.8 Sensitivity Analysis

**Fig. 4.**
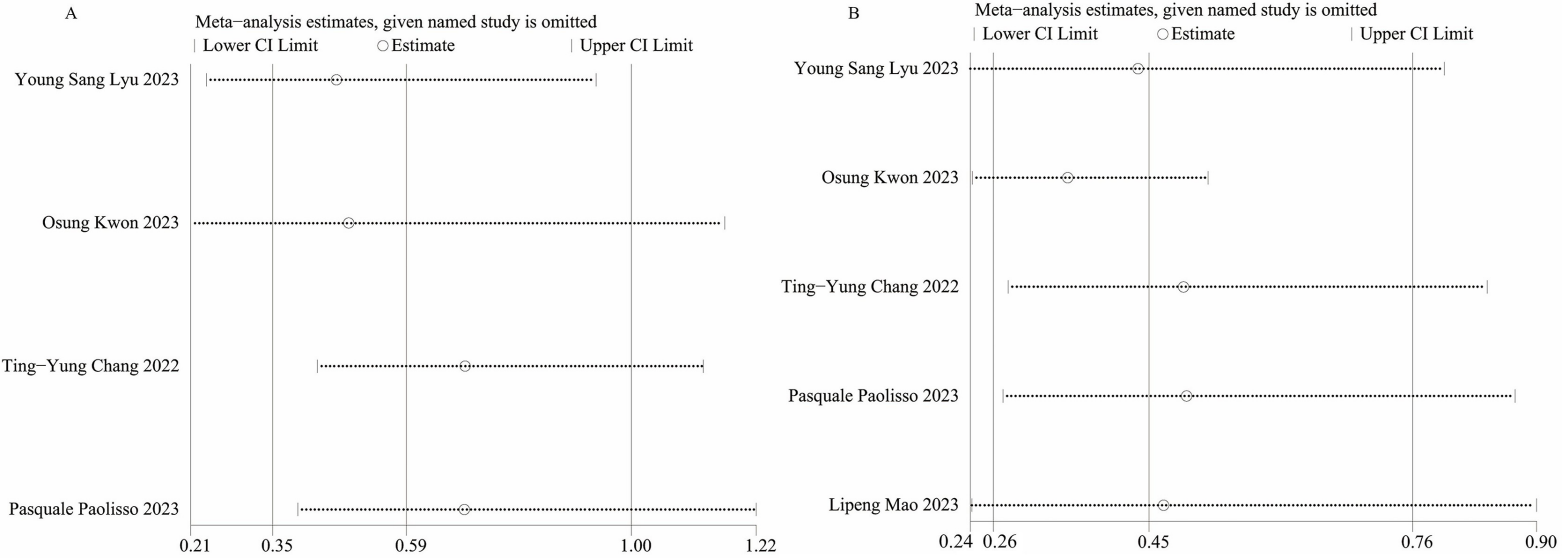
**Sensitivity analysis of (A) MACEs, (B) Rehospitalization**. MACEs, major adverse cardiovascular events.

We conducted sensitivity analyses using the leave-one-out method to evaluate the influence of each individual study on the combined OR for MACEs (Fig. 4A) and rehospitalization (Fig. 4B). Sensitivity analyses revealed that when we excluded the study conducted by Osung Kwon *et al*. [18] in 2023, the heterogeneity for rehospitalization was no longer significant (*$I^{2}$* = 0, *p* = 0.832), indicating that this study was the primary source of heterogeneity. Regarding MACEs, no sources of heterogeneity were identified. The incidence of MACEs was not statistically significant after excluding the studies by Osung Kwon *et al*. [18] (OR, 0.49 [95% CI, 0.21–1.17]; *p* = 0.11), Ting-Yung Chang *et al*. [19] (OR, 0.70 [95% CI, 0.43–1.13]; *p* = 0.14), or Pasquale Paolisso *et al*. [20] (OR, 0.70 [95% CI, 0.40–1.22]; *p* = 0.21). This result suggested that the pooled results for MACEs were not robust.

## 4. Discussion

Patients who have suffered AMI are at risk of recurrent MI, chronic heart failure, life-threatening arrhythmia, and cardiovascular death [22, 23, 24, 25]. In particular, DM patients tend to have worse prognoses after AMI [4]. The EMPA-REG OUTCOME trial showed that SGLT2-I, as a new generation of cardiorenal protective agents, can significantly improve cardiovascular mortality and reduce hospitalizations for heart failure among T2DM patients with a high cardiovascular risk [26]. However, it remains uncertain whether SGLT2-I can improve the prognosis in DM patients who experience AMI. Therefore, we performed a meta-analysis of 5 comparative studies including 5398 patients to evaluate the impact of SGLT2-I on the prognosis of DM patients who experience AMI.

This is the first meta-analysis evaluating the effects of SGLT2-I on the outcomes of DM patients who have experienced AMI. In our investigation, we found that the SGLT2-I group showed significant improvements in cardiovascular mortality and all-cause mortality compared to the non-SGLT2-I group. These findings are consistent with those reported by Faiez Zannad *et al*. [27] in their meta-analysis of heart failure patients. The precise mechanisms responsible for the beneficial effects of SGLT2-I in these patient populations have not been fully elucidated. These effects do not seem to be primarily associated with glucose control and instead appear to stem from direct cardioprotective and nephroprotective actions. These effects could be associated with various mechanisms, including the regulation of sodium balance, maintenance of energy homeostasis, reduction of cellular stress, enhancement of endothelial function, and promotion of vasodilation [28, 29, 30, 31]. Animal studies have demonstrated that SGLT2-I can lower mortality rates after AMI by altering cardiac metabolomes and elevating antioxidant levels in diabetic rats [32]. In addition, SGLT2-I also appear to have an effect in reducing the size of myocardial infarctions, enhancing left ventricular (LV) function, and lowering the incidence of arrhythmias [10], collectively contributing to improved cardiac outcomes.

Myocardial reinfarction is an important indicator in assessing prognosis. In our meta-analysis, there was no significant difference in the rate of myocardial reinfarction between the SGLT2-I and non-SGLT2-I groups. These findings are consistent with those reported by Jason H Y Wu *et al*. [33], who found that SGLT2-I did not reduce the incidences of fatal myocardial infarction or unstable angina. Importantly, among the included studies, most post-myocardial infarction patients needed one or more medications, including aspirin, P2Y12 inhibitors, beta-blockers, angiotensin converting enzyme (ACE) inhibitors or angiotensin receptor blocker (ARBs), and statins. These medications reduced the risk of myocardial reinfarction. This could be the reason why SGLT2-I do not have a statistically significant effect on reducing recurrent myocardial infarction. While not statistically significant, SGLT2-I may still have potential effects on coronary arteries. For example, Raffaele Marfella *et al*.’s [34] research indicated that SGLT2-I may have a beneficial impact on coronary artery remodeling, which is likely achieved through the regulation of a series of metabolic, molecular, and hemodynamic mechanisms that are independent of their glucose-lowering properties. Some studies have also suggested that SGLT2-I may not directly inhibit coronary thrombosis but instead focus on attenuating neurohormonal activation, minimizing cardiomyocyte necrosis, and reducing reperfusion injury [30, 35, 36, 37].

The pooled analysis of rehospitalization data indicated that SGLT2-I lowered rehospitalization rates among patients. However, the presence of significant heterogeneity was observed, potentially attributable in part to publication bias. Despite significant heterogeneity in the studies reporting data on rehospitalization, the effect remained significant when we excluded the main sources of heterogeneity (OR, 0.35 [95% CI, 0.24–0.52]; *p* = 0). These results were consistent with the findings of the meta-analysis reported by Husam M. Salah *et al*. [38], even though their study focused on patients with heart failure. For MACEs, the pooled results suggest that SGLT2-I may have a potential benefit in reducing the risk of MACEs. However, we cannot ignore that the pooled results showed significant heterogeneity (*$I^{2}$* = 71.8%, *p* = 0.01). The funnel plot reveals indications of publication bias. Sensitivity analysis further indicated that the pooled results were not stable. This heterogeneity may stem from differences in methodology, participant characteristics, treatment protocols, or other factors across studies. Thus, although we see an overall trend, the results should be interpreted with caution. Our study had multiple limitations. First, the scope of the study population was limited to T2DM patients who experienced AMI and the number of included studies was smaller than anticipated. Second, the included studies were retrospective, and there were no prospective studies to provide stronger evidence of causality. Third, due to the small number of included studies, we did not perform regression analysis to evaluate the correlation between population characteristics and clinical outcomes. Fourth, regarding the administration of SGLT2-I, the dose and duration varied across the included studies, which may have created confounding bias in the evaluation. Lastly, it is important to acknowledge the inherent constraints of meta-analyses, including heterogeneity, publication bias, variable data quality, and the absence of individual participant data, as additional limitations in our study’s methodology.

## 5. Conclusions

The pooled analyses revealed that SGLT2-I were associated with reductions in all-cause mortality, cardiovascular death, and rehospitalization. In the future, prospective studies with larger sample sizes are needed to confirm and refine these findings.

## Supplementary Material

Supplementary material associated with this article can be found, in the online version, at https://doi.org/10.31083/j.rcm2505154.
