# Supplementary material for: Effect of SGLT-2 Inhibitors on Prognosis in Diabetic Patients with Acute Myocardial Infarction: A Systematic Review and Meta-Analysis
Source: Rev Cardiovasc Med. 2024 May 6;25(5):154. doi: 10.31083/j.rcm2505154 (PMC11267217; doi:10.31083/j.rcm2505154)
Supplement: Supplementary file 1 [file 2153-8174-25-5-154-s1.zip › 2153-8174-25-5-154-s1/Supplementary Table 1.pdf]

Supplementary Table 1: search syntax

Date base	Search strategy
PubMed	(dapaglifozin OR Ertugliflozin OR empagliflozin OR Cargliflozin OR SGLT2 OR SGLT-2 Inhibitors OR SGLT 2 Inhibitors OR Sodium-Glucose Transporter 2 Inhibitor OR Sodium Glucose Transporter 2 Inhibitor OR SGLT2 Inhibitor OR Inhibitor, SGLT2 OR Gliflozins OR Gliflozin OR SGLT-2 Inhibitor OR Inhibitor, SGLT-2 OR SGLT 2 Inhibitor OR SGLT-2 inhibitor OR sodium-glucose co-transporter 2 inhibitor) AND (Cardiovascular Stroke OR Cardiovascular Strokes OR Stroke, Cardiovascular OR Strokes, Cardiovascular OR Myocardial Infarct OR Infarct, Myocardial OR Infarcts, Myocardial OR Myocardial Infarcts OR Heart Attack OR Heart Attacks OR acute myocardial infarction OR AMI OR heart attack)
Embase	('dapaglifozin' OR 'Ertugliflozin' OR ' empagliflozin' OR 'Cargliflozin' OR 'SGLT2' OR 'SGLT-2 Inhibitors' OR 'SGLT 2 Inhibitors' OR 'Sodium-Glucose Transporter 2 Inhibitor' OR 'Sodium Glucose Transporter 2 Inhibitor' OR 'SGLT2 Inhibitor' OR 'Inhibitor, SGLT2' OR 'Gliflozins' OR 'Gliflozin' OR 'SGLT-2 Inhibitor' OR 'Inhibitor, SGLT-2' OR 'SGLT 2 Inhibitor' OR 'SGLT-2 inhibitor' OR 'sodium-glucose co-transporter 2 inhibitor') AND ('Cardiovascular Stroke' OR 'Cardiovascular Strokes' OR 'Stroke, Cardiovascular' OR 'Strokes, Cardiovascular' OR 'Myocardial Infarct' OR 'Infarct, Myocardial' OR 'Infarcts, Myocardial' OR 'Myocardial Infarcts' OR 'Heart Attack' OR 'Heart Attacks' OR 'acute myocardial infarction' OR 'AMI' OR 'heart attack')
Web Of Science	(dapaglifozin OR Ertugliflozin OR empagliflozin OR Cargliflozin OR SGLT2 OR SGLT-2 Inhibitors OR SGLT 2 Inhibitors OR Sodium-Glucose Transporter 2 Inhibitor OR Sodium Glucose Transporter 2 Inhibitor OR SGLT2 Inhibitor OR Inhibitor, SGLT2 OR Gliflozins OR Gliflozin OR SGLT-2 Inhibitor OR Inhibitor, SGLT-2 OR SGLT 2 Inhibitor OR SGLT-2 inhibitor OR sodium-glucose co-transporter 2 inhibitor) AND (Cardiovascular Stroke OR Cardiovascular Strokes OR Stroke, Cardiovascular OR Strokes, Cardiovascular OR Myocardial Infarct OR Infarct, Myocardial OR Infarcts, Myocardial OR Myocardial Infarcts OR Heart Attack OR Heart Attacks OR acute myocardial infarction OR AMI OR heart attack)
Cochrane Library	((dapaglifozin) OR (Ertugliflozin) OR (empagliflozin) OR (Cargliflozin) OR (SGLT2) OR (SGLT-2 Inhibitors) OR (SGLT 2 Inhibitors) OR (Sodium-Glucose Transporter 2 Inhibitor) OR (Sodium Glucose Transporter 2 Inhibitor) OR (SGLT2 Inhibitor) OR (Inhibitor, SGLT2) OR (Gliflozins) OR (Gliflozin) OR (SGLT-2 Inhibitor) OR (Inhibitor, SGLT-2) OR (SGLT 2 Inhibitor) OR (SGLT-2 inhibitor) OR (sodium-glucose co-transporter 2 inhibitor))AND ((Cardiovascular Stroke) OR (Cardiovascular Strokes) OR (Stroke, Cardiovascular) OR (Strokes, Cardiovascular) OR (Myocardial Infarct) OR (Infarct, Myocardial) OR (Infarcts, Myocardial) OR (Myocardial Infarcts) OR (Heart Attack) OR (Heart Attacks) OR (acute myocardial infarction) OR (AMI) OR (heart attack))
